# Short-term powdered tart cherry supplementation encircling an acute endurance challenge potentially increases running performance and attenuates post-race markers of inflammation

**DOI:** 10.1186/1550-2783-12-S1-P7

**Published:** 2015-09-21

**Authors:** A O'Connor, K Levers, R Dalton, E Galvan, C Goodenough, S Simbo, S Mertens-Talcott, C Rasmussen, M Greenwood, R Kreider

**Affiliations:** 1 Exercise & Sport Nutrition Lab, Department of Health and Kinesiology, Texas A&M University, College Station, TX 77843, USA; 2 Department of Nutrition and Food Science, Texas A&M University, College Station, TX 77843, USA

## Background

Consumption of tart cherry juice has been reported to increase endurance aerobic performance and attenuate perceptions of muscle soreness by reducing inflammation and oxidative stress that cause secondary muscle damage following endurance exercise. The purpose of this study was to determine if consumption of a powdered form of tart cherries derived from tart cherry skins (CherryPURE^® ^Freeze Dried Tart Cherry Powder) prior to and following strenuous endurance exercise increases performance while attenuating markers of inflammation and oxidative stress.

## Methods

27 endurance trained or triathlete (21.8 ± 3.9 yr, 15.0 ± 6.0% body fat, 67.4 ± 11.8kg) men (n = 18) and women (n = 9) were matched based on average reported race pace, age, body weight, and fat free mass. Subjects were randomly assigned to ingest in a double blind manner capsules containing a placebo (P, n = 16) or powdered tart cherries [CherryPURE^®^] (TC, n = 11). The runners ingested the supplements one time daily (480mg/d) for 10-d: 7-d pre-exercise, day of exercise, and 48-hr post-exercise. Subjects participated in a study-organized half-marathon race (13.1mi/21.1km) at competition-pace with a 2-h (111.98 ± 11.9 min) maximum finish time. Official race splits and finish times were recorded using a standard stopwatch timing system and analyzed by a one-way ANOVA. Blood samples were drawn pre-run, 60-min post-run as well as after 24-h and 48-h of recovery and analyzed by MANOVA with repeated measures.

## Results

Significantly faster half-marathon split (p = 0.002) and race finish (p = 0.001) times were reported for subjects in the TC group versus P. The overall MANOVA analyses revealed significant Wilks' Lambda time (p < 0.001) interactions, but no significant group × time pro-inflammatory (p = 0.90) and anti-inflammatory (p = 0.73) effects. All of the univariate measures for pro- and anti-inflammatory makers reported main time effects. The mean TC IL-1β result (p = 0.059) tended to be greater than P, but no between group change over time. A trend toward a significant group × time interaction was shown in IL-2 (p = 0.079) and IL-5 (p = 0.076) with a significantly greater TC IL-2 pre-run response and a greater TC IL-5 level over all five study time points compared to P. A significant group × time interaction was shown for IL-6 (p = 0.038) and IL-12p70 (p = 0.050) with a significantly greater TC IL-6 response at baseline, pre-run, and 60-min post-run compared to P. Post-hoc analysis indicated a significantly attenuated TC IL-12p70 response across all three post-run recovery time points compared to P. The overall delta MANOVA analyses revealed significant Wilks' Lambda time (p < 0.001) interactions, but no significant group × time pro-inflammatory (p = 0.77) and anti-inflammatory (p = 0.64) effects. Within the univariate analysis, changes in IL-2 (p = 0.10), IL-5 (p = 0.067), IL-12p70 (p = 0.036), and IL-13 (p = 0.096) from pre-run tended to be more significantly attenuated in TC over P coupled with a tendency for IL-2 (p = 0.098), IL-12p70 (p = 0.098), and IL-13 (p = 0.087) levels to be more greatly attenuated in TC compared to P. The 48-h recovery NT response from pre-run tended to be greater in TC compared to P. Post-hoc analysis revealed significantly attenuated changes in IL-2 and IL-5 from pre-run values at both 24-h and 48-h post-run in TC compared to P. These results were paired with significantly attenuated IL-13 levels from pre-run at 60-min and 48-h post-run, while the NT TC response was significantly greater and the IL-12p70 TC levels were significantly attenuated compared to P at 48-h post-run. While a main delta time effect was shown for IL-6 (p < 0.001), no significant delta changes between groups over time were observed (p = 0.16).

## Conclusion

The results of the current study involving the consumption of a Montmorency powdered TC supplement for 10-d surrounding an endurance running challenge demonstrated faster race completion and a similar effect on oxidative stress and inflammation reported in previous tart cherry juice supplementation literature. Coupled with the dampening of the post-run immune and inflammatory response, the powdered tart cherry runners seemed to maintain better post run redox balance compared to placebo-supplemented runners. Further research is necessary to determine active fruit phytochemical-related inflammatory and oxidative stress mechanisms in relation to high volume endurance challenges.

